# Can angiotensin II affect the inflammatory response of human dental pulp cells *in vitro*?

**DOI:** 10.1590/1678-7765-2025-0822

**Published:** 2026-03-30

**Authors:** Bella Luna Colombini-Ishikiriama, Thiago José Dionisio, Thais Francini Garbieri, Rafaela Alves da Silva, Sandra Helena Penha Oliveira, Vanessa Soares Lara, Andrew Seth Greene, Carlos Ferreira dos Santos

**Affiliations:** 1 Universidade de São Paulo Faculdade de Medicina de Bauru Bauru Brasil Universidade de São Paulo, Faculdade de Medicina de Bauru, Bauru, Brasil.; 2 Universidade de São Paulo Faculdade de Odontologia de Bauru Departamento de Ciências Biológicas, Disciplina de Farmacologia Bauru Brasil Universidade de São Paulo, Faculdade de Odontologia de Bauru, Departamento de Ciências Biológicas, Disciplina de Farmacologia, Bauru, Brasil.; 3 Universidade de São Paulo Faculdade de Odontologia de Bauru Centro Integrado de Pesquisa Bauru Brasil Universidade de São Paulo, Faculdade de Odontologia de Bauru, Centro Integrado de Pesquisa, Bauru, Brasil.; 4 Universidade Estadual Paulista Faculdade de Odontologia de Araçatuba Departamento de Ciências Básicas Araçatuba Brasil Universidade Estadual Paulista, Faculdade de Odontologia de Araçatuba, Departamento de Ciências Básicas, Araçatuba, Brasil.; 5 Universidade de São Paulo Faculdade de Odontologia de Bauru Departamento de Cirurgia, Estomatologia, Patologia e Radiologia Bauru Brasil Universidade de São Paulo, Faculdade de Odontologia de Bauru, Departamento de Cirurgia, Estomatologia, Patologia e Radiologia, Bauru, Brasil.; 6 The Jackson Laboratory Bar Harbor Maine United States of America The Jackson Laboratory, Bar Harbor, Maine, United States of America.; 7 Universidade de São Paulo Faculdade de Odontologia de Bauru Departamento de Ciências Biológicas Bauru Brasil Universidade de São Paulo, Faculdade de Odontologia de Bauru, Departamento de Ciências Biológicas, Disciplina de Farmacologia, Bauru, Brasil.

**Keywords:** Pulp fibroblasts, Angiotensin II, Inflammatory mediators

## Abstract

Dental pulp fibroblasts respond to various stimuli, including microbial agents, endodontic materials, restorative procedures, pulp protection treatments, and inflammatory molecules. Its inflammatory response to injury involves a complex set of mechanisms that have been increasingly studied for their roles in this intricate biological process. Among these mechanisms is angiotensin II (ANG-II), the involvement of which in inflammation has been shown in several pathological conditions via its local generation and action.

**Objective:**

This study aimed to evaluate the capacity of fibroblasts from permanent and deciduous dental pulp to express inflammatory mediators when exposed to bacterial and inflammatory antigens. It also sought to assess the presence of components of the renin-angiotensin system (RAS) and the potential modulatory role of ANG-II in this response.

**Methodology:**

Fibroblasts were cultured from the pulp of permanent and deciduous teeth obtained from three adult and four pediatric donors with informed consent. Cells were stimulated with *Porphyromonas gingivalis* and *Escherichia coli* lipopolysaccharides (LPS) either alone or in combination with ANG-II and, in some cases, with IL-1β. Gene expression of RAS components and various inflammatory mediators was analyzed by qRT-PCR. ANG-II receptor expression was quantified by flow cytometry.

**Results:**

The results showed that both bacterial antigens and IL-1β significantly upregulated the expression of genes encoding key inflammatory mediators. However, ANG-II, either alone or in combination with other antigens, failed to alter the expression levels of these mediators.

**Conclusion:**

These findings clearly show that ANG-II has no influence on the expression of inflammatory mediators by human dental pulp fibroblasts under the tested conditions.

## Graphical abstract

**Figure:**
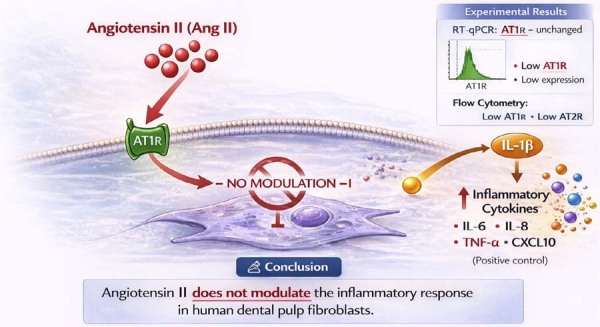

## Introduction

Dental pulp, a mesenchymal-derived tissue, harbors several resident cell types, undifferentiated precursors, and migratory/inflammatory cells within the mineralized tissue of the pulp chamber and root canals. Among these, fibroblasts stand out as the most abundant resident cell type. These cells are widely recognized for their role in producing, remodeling, and maintaining the extracellular matrix—functions typical of connective tissue cells.^1^

However, permanent and deciduous human teeth and their pulpal cells are frequently exposed to external damage, whether bacterial, from dental materials in various pulp therapies, or due to physical injuries such as trauma or iatrogenic damage. In all these situations, the pulp tissue is challenged to mount a defense response in which fibroblasts play an important role.^2-7^

Studies using pulp cells from permanent human teeth have shown that the fibroblasts from these tissues can produce an inflammatory response when stimulated by cytokines or pulp capping materials.^1,8-15^ Moreover, these cells produce several inflammatory mediators in response to bacterial components^4,16-18^ and can modulate immune cell differentiation in co-culture systems.^1^

Fibroblasts from human deciduous teeth have also shown a potential role in inflammatory responses. Studies have found that these cells significantly contribute to cytokine and chemokine production in response to non-bacterial stimuli (such as pulp capping materials) by producing IL-1β and IL-8^3^ and to bacterial stimuli by producing CCL3 and CXCL12.^17,18^ More recently, our research group evaluated the expression and production of cytokines and chemokines by these cells in response to bacterial antigens, finding that they could express genes and secrete 12 out of the 18 evaluated cytokines/chemokines. These findings reinforce the role of deciduous pulp cells in the inflammatory process.^19^

Nonetheless, the inflammatory response of the pulp is part of a broader and more complex set of events that includes vascular, cellular, and molecular mechanisms. One such mechanism is mediated by angiotensin II (ANG-II), the effector molecule of the renin-angiotensin-aldosterone system. Numerous studies have shown the pro-inflammatory effects of ANG-II in various human diseases, particularly systemic conditions such as hypertension, and in local pathologies affecting oral tissues, such as periodontal disease.^20-24^

Animal and human studies have shown the presence and activity of the renin-angiotensin system in gingival tissue and the periodontal ligament and its role in periodontal disease. Specifically, the pharmacological blockade of the ANG-II receptor (AT1R) has been shown to reduce the production of inflammatory mediators in gingival tissues.^20-25^ These studies have also shown gene expression for all components of the renin-angiotensin system, including angiotensinogen, ACE, ACE-2, AT1R, and Mas receptor by human gingival and periodontal ligament fibroblasts.^22,23^

Considering the potential of ANG-II to influence inflammatory responses in periodontal disease, could ANG-II also play a role in modulating the inflammatory response of isolated pulp fibroblasts? Moreover, when combined with bacterial antigens (LPS) and IL-1β, could it modify or amplify the inflammatory activity of these cells?

### Objectives

To address these questions, this study aimed to investigate the presence of renin-angiotensin system (RAS) receptors in cells derived from the pulp tissue of human deciduous and permanent teeth. Furthermore, we sought to assess whether ANG-II can induce an inflammatory response in pulp cells in the absence and in the presence of bacterial antigens. Additionally, we evaluated the role of IL-1β in inducing the synthesis and expression of ANG II receptors and the impact of this expression on the production of inflammatory mediators by fibroblasts.

## Methodology

### Primary cell culture

Primary cultures of human fibroblasts derived from dental pulp tissues were established via an explant technique^18,19^ using teeth extracted from three systemically healthy adult donors (two females and one male aged 22-25 years) and four children (two girls and two boys aged 7-11 years). All tissues were obtained with informed consent from patients and/or their legal guardians via signed informed consent forms, and only after approval by the Research Ethics Committee of the Bauru School of Dentistry, University of São Paulo (CAAE: 44739015.0.0000.5417).

Cells were cultured in Dulbecco’s Modified Eagle Medium (DMEM) (Invitrogen, Life Technologies Corp., Carlsbad, CA), supplemented with 10% fetal bovine serum (FBS) (Gibco, Invitrogen, Carlsbad, CA) and antibiotics (penicillin 100 mg/mL, streptomycin 100 mg/mL, and amphotericin B 0.5 mg/mL; Invitrogen). Cultures were maintained at 37 °C in a humidified atmosphere containing 5% CO₂ and 95% air. Cells between passages 4 and 8 were used in all experiments.

### Characterization of primary cell cultures

Primary pulp-derived cells from permanent and deciduous teeth were characterized as fibroblasts based on their typical spindle-shaped morphology and positive immunofluorescence staining for fibroblast surface protein (FSP) as previously described.^19^

### Cell viability assay

Cell viability and the potential cytotoxicity of the experimental challenges were evaluated using the AlamarBlue^®^ Cell Viability Reagent (Invitrogen™, Ambion, Thermo Fisher Scientific, Waltham, MA) following the manufacturer’s protocol.

### Cell stimulation

Pulp cells from deciduous teeth (n=4 donors) and permanent teeth (n=3 donors) were plated in triplicates in 24-well plates at a density of 5 × 10⁴ cells per well in DMEM supplemented with 10% FBS. After overnight adhesion, cells were incubated either with the medium alone (DMEM + 1% FBS, control group) or challenged with bacterial lipopolysaccharides—*Escherichia coli* LPS (EcLPS, 1 μg/mL; L4391, Sigma-Aldrich, St. Louis, MO) or Angiotensin II (ANG-II, 1 μM; Sigma-Aldrich^®^)—alone or in combination for six and 24 hours in the deciduous tooth-derived cell cultures. In the cultures from permanent teeth, *Porphyromonas gingivalis* LPS (PgLPS, 1 μg/mL; L4391, Sigma-Aldrich, St. Louis, MO) was used as the bacterial antigenic stimulus, with analysis performed after 24 hours.

To assess the effect of IL-1β pre-stimulation on ANG-II receptor expression, cells from the permanent and deciduous teeth were seeded in triplicates at 2 × 10⁵ cells per well in six-well plates in basal DMEM. After overnight attachment, the culture medium was replaced with DMEM containing reduced serum (1% FBS). After 24 hours of serum reduction, the cells were exposed to ANG-II (1 μM, Sigma-Aldrich^®^) for three, six, and 24 hours or to IL-1β (0.1 ng/mL; PeproTech^®^) for 24 hours either alone or followed by ANG-II stimulation for three, six, and 24 hours.

At the end of each experimental period, culture supernatants were removed, and the cells were lysed using the lysis buffer provided in the RNA isolation kit. Lysates were stored at –80 °C until RNA extraction.

### Gene expression analysis

The quantitative gene expression analysis of RAS components (AT1R, and AT2R) and a panel of cytokines and chemokines—including IL-1α, IL-1β, IL-2, IL-4, IL-6, IL-8, IL-10, IL-12, IL-17, MCP-1 (CCL2), MIP-1α (CCL3), CXCL12, RANTES (CCL5), IFN-γ, TNF-α, VEGF, and M-CSF was performed using real-time quantitative reverse transcription polymerase chain reaction. RPL13A was used as the reference gene.

RNA was extracted from cell lysates using an automated pipetting system (Nimbus, Hamilton, USA) and the MagMAX™ mirVana™ Total RNA Isolation Kit (Thermo Fisher Scientific, USA; #A27828). RNA concentration (ng/µL) and purity (A260/280 ratio) were determined spectrophotometrically using NanoDrop 1000 (Thermo Scientific, Wilmington, USA).

**Figure 1:**
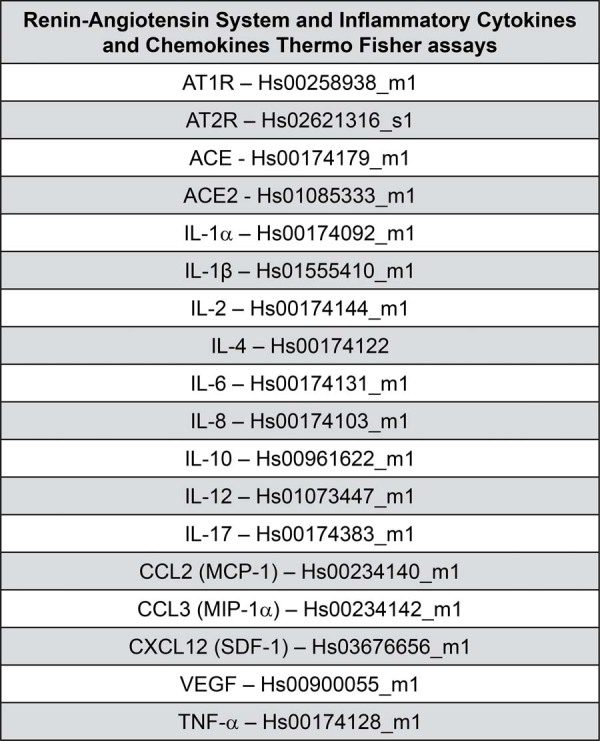
Target genes used in RT-qPCR reactions for HPF and HPFD, with respective TaqMan® Assay IDs.

Reverse transcription of total RNA into complementary DNA was performed using the High-Capacity cDNA Reverse Transcription Kit (Thermo Fisher Scientific, USA; #4368813). The quantitative PCR analysis of gene expression was then performed for the selected targets (see Figure 1) using the ViiA™ 7 Real-Time PCR System (Applied Biosystems™). Relative quantification was determined using the comparative cycle threshold method as previously described.^23^

### Presence of AT1R and AT2R receptors by flow cytometry

Immunophenotyping of AT1 and AT2 receptors (Santa Cruz Biotechnology, Dallas, Texas, USA, Cat. No. sc-515884 PE and sc-518054 PE; 1:100 dilution) was performed by flow cytometry using the BD FACSAria™ Fusion Cell Sorter (BD Biosciences, San Jose, CA, USA), and data were analyzed on FlowJo™.

### Statistical analysis

Statistical analyses were conducted on GraphPad Prism 9 (GraphPad Software, LLC, San Diego, CA, USA). Data normality was assessed by the Shapiro-Wilk test. Considering the variable stimulus times and types, two-way analysis of variance was used to detect significant differences. The Tukey’s post-test was used. Statistical significance was set at 95% confidence level (p<0.05).

## Results

### Characterization of primary cell cultures

**Figure 2:**
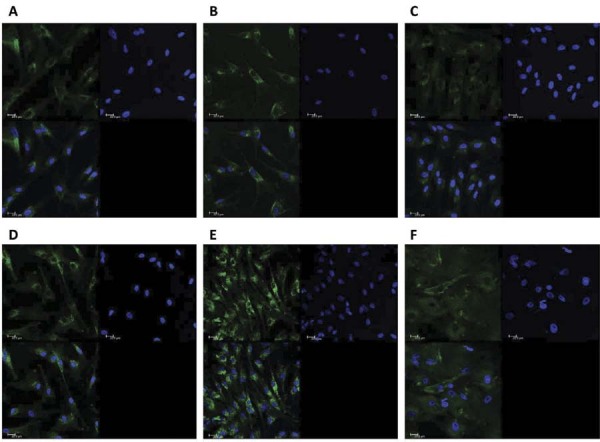
Phenotypic characterization of pulp cells by fibroblast surface protein (FSP) staining. HPF (A, B, and C) and HDPF (D, E, and F) showed positive staining for FSP-1 protein (green). Cell nuclei were stained with DAPI- blue (4’,6- diamidino-2-phenylindole dihydrochloride). Negative controls are also shown. Images were acquired using confocal microscopy (TCS model, SPE, Leica®, Mannheim, Germany) with a 40× objective lens. Scale bars represent 20 μm.

Fibroblasts from permanent and deciduous dental pulp were isolated and cultured following previous protocols.^4,17-19^ The fibroblastic phenotype of the cells was confirmed by positive staining for fibroblast surface protein-1, as shown in Figure 2.

### mRNA expression of RAS components

**Figure 3:**
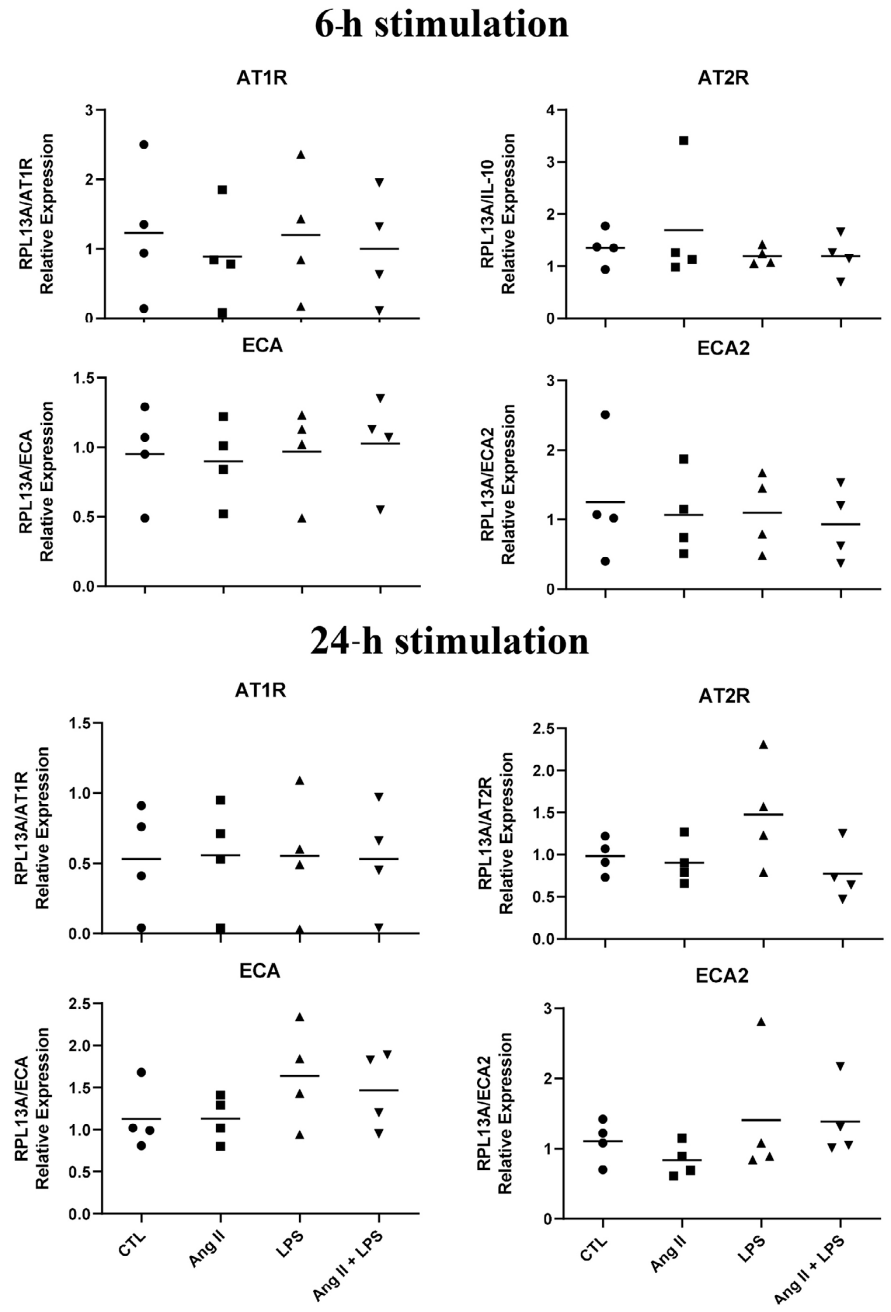
qPCR analysis of RAS components by HDPF stimulated without (Control) or with lipopolysaccharide from *Escherichia coli* (EcLPS) (1μg/mL) or ANG II (1 μM) for six and 24 hours. Relative expression levels of the target mRNA relative to RPL13 mRNA from four donors in triplicates (n = 4) are shown in graphs. The mean values for each patient are represented as a symbol to compare the stimulated values with their respective control group in the same experimental period by the non-paired t test. * indicates significant difference with the respective control in the same experimental period (p<0.05).

Gene expression results showed that cells from pulp deciduous teeth of all donors expressed the genes for the components of the renin-angiotensin system. This expression suffers no influence from exposure to ANG-II and/or LPS (Figure 3).

### mRNA expression of inflammatory mediators in deciduous pulp cells

The gene expression, evaluated by mRNA quantification, of the studied inflammatory mediators showed a highly heterogeneous regulation profile in deciduous pulp fibroblasts. Some of the cytokines assessed were not detected in any of the experimental conditions, others showed inconsistent expression, whereas some were constitutively expressed. The regulation of their expression by the studied stimuli also occurred in a heterogeneous manner. The expression patterns for each stimulus in fibroblasts from deciduous pulp can be better observed in Figures 4A  and 4B.

### mRNA expression of inflammatory mediators by IL-1β stimulation in permanent pulp cells

Stimulation with ANG-II alone failed to modulate the gene expression of the studied targets, as previously observed. However, stimulation with IL-1β positively regulated gene expression for all studied cytokines. This effect underwent no alteration by additional stimulation with ANG-II at any of evaluated timepoint (three, six, or 24 hours) in cells derived from permanent pulp (Figures 5A and5B).

### Presence of AT1R and AT2R receptors by flow cytometry

ANG-II AT1 and AT2 receptors were expressed in all the primary cultures of human pulp cells in this study. However, only a small percentage of cells expressed these receptors, ranging from 0.35 to 2.16% (mean 1.31%) for AT1, from 0.69 to 5.90% (mean 2.59%) for AT2 in permanent cells, from 2.43 to 4.75% (mean 3.48%) for AT1, and from 2.48 to 3.29% (mean 2.83%) for AT2 in deciduous cells.

## Discussion

Several studies have shown, by experiments with human dental pulp tissue cells, the potential of fibroblasts—the predominant cells of the connective pulp tissue in permanent and deciduous teeth—to respond to various stimuli. These stimuli range from bacterial antigens and the dental materials in pulp therapy to inflammatory molecules, producing multiple signaling molecules in the inflammatory process. Fibroblasts can also modulate the differentiation of immune cells, participating in the initiation and amplification of the pulpal inflammatory response.^1,4,9-19,24^

This study reconfirmed the ability of fibroblasts from primary cultures of human permanent and deciduous dental pulp tissue to produce inflammatory mediators in response to various agents. The used bacterial antigens—*P. gingivalis* and *E. coli* LPS—stimulated gene expression, producing several inflammatory mediators by fibroblasts derived from deciduous and permanent pulp tissues, respectively. IL-1β stimulation also positively regulated gene expression for signaling molecules in inflammation, which contribute to the recruitment and activation of other resident cells in the pulp tissue and the immune cells in blood vessels, which are fundamental for an adequate pulpal defense response.^7,14,26-28^

In this study, the deciduous pulp cells were studied separately from permanent pulpal cells because studies on the behavior of pulp tissues or even in cultured cells from deciduous pulps are scarce in the literature. Interestingly, previous studies by our group have shown differences in chemokine release by deciduous pulp fibroblasts when compared to fibroblasts from other oral sites,^17-18^ which justifies the comparison between deciduous and permanent teeth pulp cells in this study.

Another focus of this study was to investigate whether fibroblasts from deciduous and permanent teeth express key RAS components, such as AT1R and AT2R, and how ANG-II (the effector molecule of RAS) might influence the inflammatory potential of pulpal fibroblasts in culture. This aimed to understand whether ANG-II, which has been shown to participate locally in several inflammatory diseases (including those affecting oral tissues) could also modulate the pulpal inflammatory response. ^21,23,24,28-32^

Contrary to expectations, the inflammatory capacity of fibroblasts from primary cultures of deciduous and permanent teeth underwent no modulation due to ANG-II. Despite its pro-inflammatory potential in various pathologies, ANG-II was unable to affect the response of pulpal fibroblasts, even when combined with other inflammatory stimuli such as IL-1β or LPS from *E. coli* and *P. gingivalis*. These results are likely due to the low expression of ANG II receptors (AT1R and AT2R) in these cells. Data from flow cytometry analysis of cells from seven donors—three permanent and four deciduous teeth from female and male volunteers—showed that only a small subset of cells expressed these receptors, with AT2R receptors found in a slightly higher percentage of cells, although this was statistically insignificant.

In this context, despite the low number of ANG-II receptors- expressing cells under our experimental conditions, receptor expression could be inducible in cells that were negative in this study, as suggested by previous findings.^33^ This modulation could increase the percentage of cells that respond to ANG-II, potentially generating a significant response that was absent under the conditions of this study.

However, no other study in the literature has evaluated the presence of RAS components in fibroblasts derived from primary pulp cultures of permanent and deciduous teeth or the role of ANG-II on the expression and production of inflammatory mediators by these cells. Therefore, the results in this study are important for ruling out the possibility of ANG-II acting as a modulator of pulpal inflammation in both dentitions under conditions resembling those tested in this study. These findings direct future research toward alternative molecular pathways in the activation of the pulpal immune response and a deeper understanding of those described in the literature.

## Conclusion

The results of this study confirm that pulpal fibroblasts from permanent and deciduous teeth can be stimulated to produce inflammatory mediators in response to bacterial and other inflammatory stimuli. However, they fail to support the hypothesis that ANG-II plays a role in modulating this response, even when combined with other pro-inflammatory agents.

**Figure 4A:**
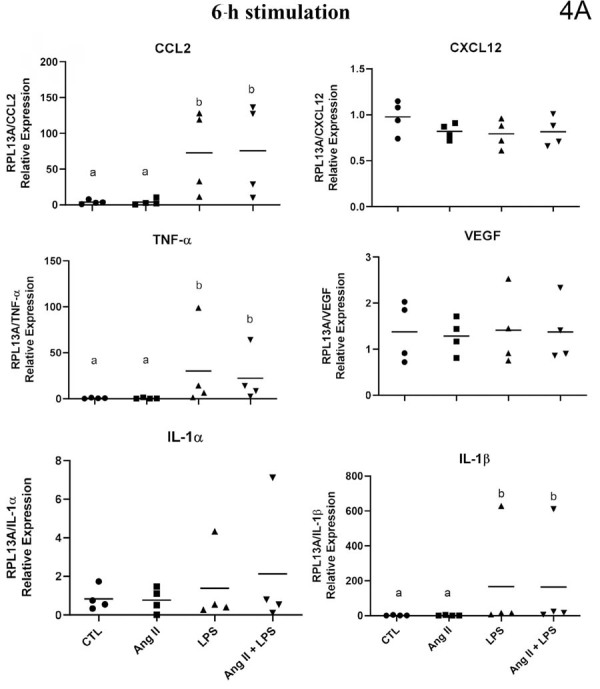
qPCR analysis of cytokines/chemokines by human deciduous dental pulp fibroblasts (HDPF) stimulated with or without (Control) lipopolysaccharide from *Escherichia coli* (EcLPS) or angiotensin II (ANG II, 1 μM) for six (4A) and 24 hours (4B). Relative expression levels of the target mRNA normalized to RPL13 mRNA from four donors in triplicates (n = 4) are shown in graphs. The following targets were analyzed: CCL2, CXCL12, TNF-α, vascular endothelial growth factor (VEGF), interleukin-1α (IL-1α), and interleukin-1β (IL-1β). Mean values for each donor are represented by individual symbols. Expression levels in stimulated groups were compared to their respective control group at the same time point using an unpaired t-test. Asterisks indicate statistically significant differences to the respective control group at the same time point (p<0.05).*

**Figure 4B:**
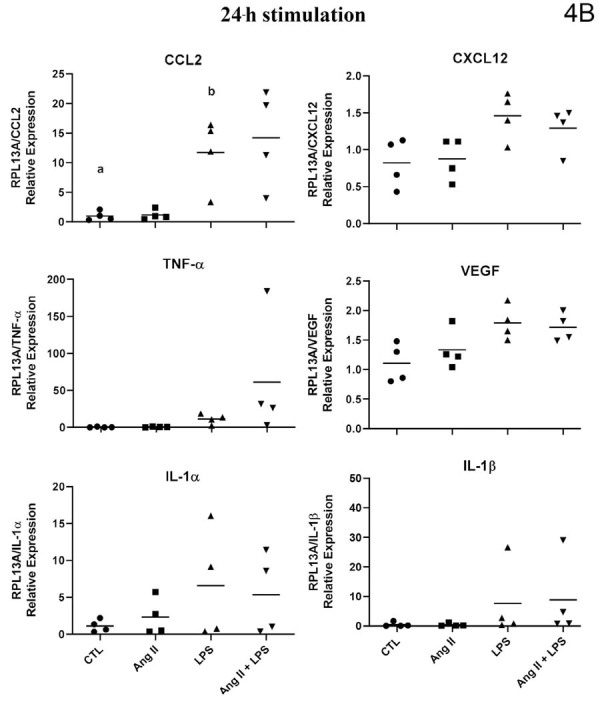
qPCR analysis of cytokines/chemokines by human deciduous dental pulp fibroblasts (HDPF) stimulated with or without (Control) lipopolysaccharide from *Escherichia coli* (EcLPS) or angiotensin II (ANG II, 1 μM) for six (4A) and 24 hours (4B). Relative expression levels of the target mRNA normalized to RPL13 mRNA from four donors in triplicates (n = 4) are shown in graphs. The following targets were analyzed: CCL2, CXCL12, TNF-α, vascular endothelial growth factor (VEGF), interleukin-1α (IL-1α), and interleukin-1β (IL-1β). Mean values for each donor are represented by individual symbols. Expression levels in stimulated groups were compared to their respective control group at the same time point using an unpaired t-test. Asterisks indicate statistically significant differences to the respective control group at the same time point (p<0.05).*

**Figure 5A:**
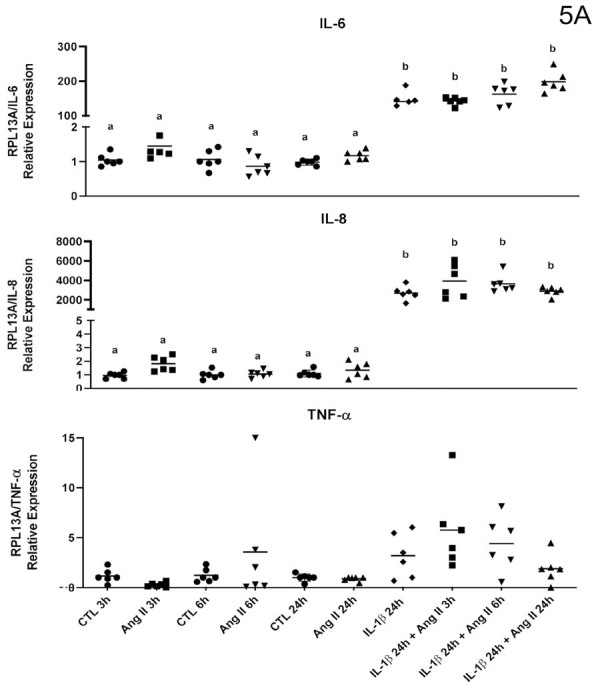
qPCR analysis of cytokines/chemokines by human dental pulp fibroblasts (HPF) stimulated with or without (Control) angiotensin II (ANG II, 1 μM) or interleukin-1β (IL-1β, 0.1 ng/mL) in isolation or combined for three, six, and 24 hours. Relative expression levels of the target mRNA normalized to RPL13 mRNA from three donors in triplicates (n=3) are shown in graphs. The following targets were analyzed: IL-6, IL-8, TNF-α (5A), CCL2, COX-2, and AT1R (5B). Mean values for each donor are represented by individual symbols. Expression levels in stimulated groups were compared to their respective control group at the same time point using an unpaired t-test. Asterisks indicate statistically significant differences to the respective control group at the same time point (p<0.05).

**Figure 5B:**
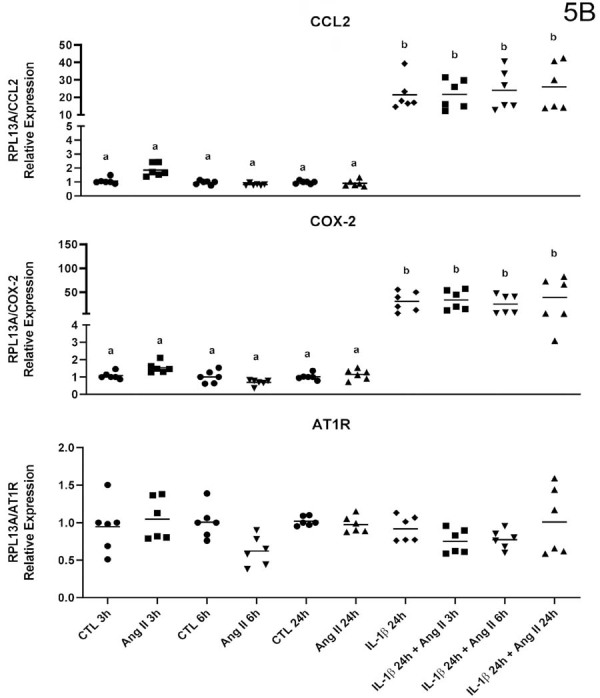
qPCR analysis of cytokines/chemokines by human dental pulp fibroblasts (HPF) stimulated with or without (Control) angiotensin II (ANG II, 1 μM) or interleukin-1β (IL-1β, 0.1 ng/mL) in isolation or combined for three, six, and 24 hours. Relative expression levels of the target mRNA normalized to RPL13 mRNA from three donors in triplicates (n=3) are shown in graphs. The following targets were analyzed: IL-6, IL-8, TNF-α (5A), CCL2, COX-2, and AT1R (5B). Mean values for each donor are represented by individual symbols. Expression levels in stimulated groups were compared to their respective control group at the same time point using an unpaired t-test. Asterisks indicate statistically significant differences to the respective control group at the same time point (p<0.05).
